# Observational Study on the Management of Children with Systemic Diseases During Dental Treatment

**DOI:** 10.3390/children13050701

**Published:** 2026-05-20

**Authors:** Tatsuya Akitomo, Satoru Kusaka, Ryota Nomura

**Affiliations:** 1 Department of Pediatric Dentistry, Graduate School of Biomedical and Health Sciences, Hiroshima University, Hiroshima 734-8553, Japan; rnomura@hiroshima-u.ac.jp; 2 Department of Pediatric Dentistry, Hiroshima University Hospital, Hiroshima 734-8551, Japan; higechi@hiroshima-u.ac.jp

**Keywords:** dental procedure, pediatric dentistry, systemic disease

## Abstract

**Background/Aim**: While improvements in oral health can sometimes lead to improvements in systemic diseases, certain systemic diseases such as heart disease require consideration during dental treatment. In clinical pediatric dentistry, dental professionals may encounter the pediatric patient with systemic diseases, and should consult with physicians regarding the overall health status of their patients with systemic diseases before dental procedures. **Materials and Methods**: We reviewed the responses to letters of inquiry made to external medical institutions regarding the overall physical condition of patients during a 5-year period (2021–2025). The survey items were the gender, age, aim of letter, systemic disease, and physician responses and instructions. **Result**: A total of 48 letters of inquiry were found, of which 34 were initial confirmations and 14 were reconfirmations. In children aged ≤5 years, the common aim was oral management or caries treatment, and the proportion of surgical procedures increased with age. Among the systemic diseases involved, the diseases of the circulatory system such as congenital heart disease were the most common, with 70% of initial confirmations leading to premedication with antibiotics prior to invasive dental procedures. Although many responses remained unchanged upon reconfirmation, the responses for some patients changed. **Conclusions**: This study shows the importance of dental professionals consulting with physicians regarding the health condition of patients with systemic diseases before oral management. Furthermore, a single confirmation is insufficient, and the information must be regularly updated.

## 1. Introduction

In recent years, oral diseases have been reported to be associated with systemic diseases. In type 2 diabetes mellitus, the association between periodontal disease and its development is well established, and some studies have shown that management of periodontal disease may improve glycemic control [1]. Periodontitis also increases the risk of cerebrovascular disease and dementia, and various oral health interventions can reduce the future risk [2]. On the other hand, it has been reported that Streptococcus mutans, a major pathogen of dental caries, can exacerbate various systemic diseases in strains that leads to production of Cnm (collagen-binding cell surface protein) [3]. With the growing interest in oral hygiene, dental professionals frequently encounter patients with systemic diseases.

Although this is also true in pediatric dentistry, pediatric patients have various chief complaints. Even if efforts are made to prevent oral diseases, surgical dental treatment may be necessary because of tooth replacement, tooth impaction, supernumerary teeth, and dental trauma. Various systemic diseases, including heart diseases, require consideration during dental treatment. Individuals with heart conditions, such as artificial heart valves or congenital heart diseases, are at risk of developing infective endocarditis, a rare but life-threatening infection [4]. The American Heart Association (AHA) issued its first statement on the prevention of infective endocarditis in 1955, and the guidelines have since been updated during the last two decades to restrict the use of antibiotic prophylaxis to the highest-risk patients [4,5]. The Japanese Circulation Society (JCS) guidelines state that the use of antibiotic prophylaxis depends on various risk factors for infective endocarditis, such as the heart condition and the type of dental procedure [6]. However, the heart condition varies among patients, and dental professionals cannot judge the condition of all patients based on diagnosis alone. Therefore, it is important to consult a physician to review the relevant points to keep in mind when undergoing dental procedures. A need for premedication for infective endocarditis has been reported, and a new system for sharing medical information in Japan’s health insurance, called “medical information sharing fee”, was established in 2018, making it covered by insurance for dentists to check a patient’s systemic condition with their physician [7]. However, few studies have investigated the contents of instructions given by physicians, and many aspects of this situation remain unknown. In this study, we investigated letters of inquiry written to physicians for patients with systemic diseases and examined the recommended precautions to take during dental treatment.

## 2. Materials and Methods

### 2.1. Subjects

This study was approved by the Hiroshima University Epidemiology Research Ethics Committee (approval number: E2025-0234; date: 29 January 2026), and patient consent was waived due to a single-center retrospective observational study using only existing medical records. The protocol is reported in line with the Strengthening the Reporting of Observational Studies in Epidemiology (STROBE) statement [8].

Almost 7000 pediatric patients, including 500 new pediatric patients, are treated in the Department of Pediatric Dentistry at Hiroshima University Hospital every year. In 2018, the Japanese national insurance system was updated to enable dentists to write letters of inquiry to physicians to better understand the overall health condition of their patients. The present study examined patients who were the subjects of letters of inquiry from our department to physicians at other medical institutions during the 5-year period from 2021 to 2025. The study used an observational study design and subjects who met the eligibility criteria described below.

### 2.2. Inclusion Criteria

The inclusion criteria for this study included the following:

- Patients for whom medical information sharing fees were charged from 2021 to 2025.
- Patients who have written an inquiry letter to their physician.

### 2.3. Exclusion Criteria

The exclusion criteria for this study included the following:

- Patients who could not receive a reply.
- Patients with insufficient medical information for this study.

### 2.4. Survey Items

Using medical records, we extracted information on gender, age at time the letter of inquiry was written, aim of letter, systemic disease, and physician responses and instructions. Systemic diseases were classified in accordance with the International Classification of Diseases the 11th version, and those with few cases were included under “Others” [9]. Although the formats of the inquiry letters and items to confirm were standardized, the wording was not exactly identical down to every single word because multiple dentists were involved. Therefore, we investigated what kind of dental treatment the dentists are currently planning for the patient, and it was defined as the aim of the inquiry letter. The aims were subsequently classified into “oral management”, “caries treatment”, and “surgical procedure”. In brief, “oral management” includes patients who do not have any planned dental treatments other than oral disease prevention, and who have confirmed to keep this in mind during regular dental checkups. On the other hand, patients scheduled for caries treatment were classified as “caries treatment”, while those scheduled for surgical dental approach, such as extraction of primary teeth or supernumerary teeth, were classified as “surgical procedures”.

All inquiry letters were sent to the physician who was examining the patient’s systemic condition that the dentist wanted to confirm. Responses to the first letter from the physician were counted as “first confirmation”, while responses regarding the patient who had previously received responses from physicians were counted as “reconfirmation”, and we compared the previous instructions with the new instructions.

## 3. Results

A total of 52 letters of inquiry were found over the 5-year period and 2 were excluded due to insufficient information. Of 50 letters, 48 received replies and were included in the study. The response rate of 96%. The average number of days from the creation of the inquiry letter to the physician’s creation of a reply was 8.8 days, and the median was 6.0 days. In addition, the average number of days from the creation of the inquiry letter to incorporating it into the medical record was 22.5 days, and the median was 17.5 days.

The changes in the letters of inquiry over time are shown in Table 1. Among the 48 letters, 5 letters involved the same patients (one male patient and four female patients). Therefore, the total number of patients was 43 (20 male patients and 23 female patients). Although the number of letters varied per year, the number showed a continuous increase from 2023 to 2025. The ages and aims at the time of inquiry letter creation are shown in Table 2. Of the 48 letters, 34 were first confirmation letters, and 14 were reconfirmation letters. For the first confirmation letters, the most common age group was 0–5 years and 6–8 years, followed by 9–11 years, with most letters involving patients below 11 years of age. The most common aim of the first confirmation letters was oral management and caries treatment in patients aged 0–5 years, while surgical procedures became more common in older patients. For the reconfirmation letters, the most common age group was 9–11 years, followed by 12–15 years. Younger age was more likely to be associated with the aim of oral management, while surgical procedure increased as an aim after 9 years of age.

Among the 34 first confirmation letters, more than 70% involved patients with diseases of the circulatory system, followed by patients with diseases of the nervous system, diseases of the immune system, and diseases of the ear or mastoid process (Table 3). Most of diseases of the circulatory system were congenital heart defects, such as ventricular septal defects. Among the patients with diseases of the circulatory system, 70% required antibiotics before invasive dental procedures. Some patients with diseases of the nervous system had things to be careful about when dental treatment was performed under body restraint. For the patients with diseases of the immune system, especially those undergoing sublingual immunotherapy, opinions were divided in the responses. One physician instructed that sublingual immunotherapy should be discontinued for a certain period after the surgical procedure, while another physician recommended that treatment should be continued while keeping the medicine away from the wound.

Most of the 14 reconfirmation letters involved patients with diseases of the circulatory system (Table 4). As was the case in the initial confirmation, congenital heart disease accounted for a large proportion of diseases of the circulatory system. For these patients, more than 80% received the same response as the preceding response, with no changes, while one patient each required antibiotics for an invasive dental procedure and no longer required antibiotics.

## 4. Discussion

Medical and dental illnesses are often associated, underscoring the importance of integrating a patient’s medical and dental care [10]. Although dentists are ultimately responsible for the treatment they provide, it is important to obtain complete medical information about their patients and undertake consultations with physicians when planning dental procedures [10]. While physicians’ responses vary depending on the patient, dentists need to understand what kinds of responses might require consideration in different situations. However, there are very few reports that focus on responses received to inquiries made to medical doctors about a patient’s overall physical condition. Understanding what instructions may be given depending on a patient’s overall condition provides dentists with important guidance for performing dental treatment safely for patients. This is where the clinical significance of this study lies, and we investigated the management of children with systemic diseases for whom information was requested from physicians. Over a 5-year period, 48 letters of inquiry were identified. The number of such letters was very low, at approximately 10 per year. This may be related to the fact that our hospital has a medical department, and thus, dentists can obtain access to the electronic health records without written confirmation when patients have a pediatrician in the hospital.

The physicians prepared a reply about a week after receiving the letter of inquiry, and the dentist confirmed the contents about three weeks later, which shows that physicians are responding to pediatric patients as quickly as possible. On the other hand, the time it takes to import medical records may have been slightly longer because it includes cases where the patient brings the reply form to their next appointment, as well as the time required to mail the reply and for the dentist to process its import after receiving it. Dental professionals should know that it takes about three weeks from the time an inquiry letter is issued to fully understand the patient’s systemic condition.

Of the 48 letters of inquiry, 34 were first confirmation letters sent to physicians. The common ages for the children were 0–5 years and 6–8 years, and the common aims for the consultation in 0–5 years were oral management and caries treatment. In contrast, the reconfirmation letters predominantly involved students in the middle to upper grades of elementary school, and the aim was surgical dental procedures. Traumatic dental injuries are common in children and are frequently encountered in general dental practice [11]. A previous study indicated that more than 40% of children had experienced dental trauma by 4 years of age, with timely and effective treatment being crucial for the management of dental trauma when dental injuries occur [12]. However, as mentioned above, it takes about three weeks to respond to an inquiry letter. In emergencies, such as when a child with immunodeficiency, thrombocytopenia, or heart disease suffers a dental trauma, there are time constraints. Pediatric patients with systemic disease require a collaboration between the physician and dentist with or without trauma [13]. Therefore, when a pediatric patient with a systemic disease presents at a clinic, it is advisable to consult their physician regarding the patient’s systemic condition before performing any oral care, even if no obvious pathological findings are present. Moreover, pediatric patients undergo a period of permanent tooth eruption during which surgical procedures, such as primary tooth extraction, may be necessary. Therefore, it is advisable to assess the overall health status during the tooth replacement period.

The systemic diseases for which information was requested most frequently were diseases of the circulatory system such as congenital heart diseases, for both the first confirmation letters and the reconfirmation letters. As mentioned above, the AHA recommended in 1955 that antibiotic prophylaxis should be used to prevent infective endocarditis, and their recommendation included “all subjects with rheumatic or congenital heart disease undergoing dental extractions and other dental manipulations which disturb the gums, the removal of tonsils and adenoids, the delivery of pregnant women, and operations on the gastrointestinal or urinary tracts” [4,5]. Subsequently, the AHA, the European Society of Cardiology (ESC), and the National Institute for Health and Care Excellence each recommended varying degrees of restriction on antibiotic prophylaxis, with both the AHA and the ESC recommending that antibiotic prophylaxis should only be considered for the highest-risk patients [4]. Thornhill et al. performed the 9.6 million patient cohort and concluded the risk of infective endocarditis was high in high-risk individuals following all types of invasive dental procedures [14]. Moreover, numerous meta-analyses have shown that invasive dental procedures can cause bacteremia, which is a risk factor for infective endocarditis in high-risk individuals [15,16]. In Japan, JCS released the first edition of its guidelines for the prevention and treatment of infective endocarditis in 2003, and these guidelines were subsequently revised in 2008 and 2018 [6]. In 2026, the JCS guideline was updated, and it recommends antibiotic prophylaxis for the highest-risk patients when all invasive dental procedures that cause bleeding and bacteremia [6]. In this study, 70% of patients were considered high risk after consultation with their attending physicians. Although the necessity for premedication should be determined based on the cardiac risk and the nature of the dental procedure, the present findings suggest that a significant number of pediatric patients require special consideration when undergoing dental procedures. However, a previous study reported that guideline adherence was not necessarily high [6]. It is important to deepen the knowledge regarding the prevention of infective endocarditis and to appropriately consult with physicians when such patients visit dental clinics. On the other hand, antibiotic overuse is associated with antimicrobial resistance (AMR), a critical public health issue [17,18]. It is essential to ensure the appropriate use of antibiotics to prevent their overprescription and, consequently, to avoid contributing to the development of AMR.

This study also compared the responses received from consulting physicians to the reconfirmation letters sent after several years with their responses to the first confirmation letters. The results revealed that the responses remained largely unchanged in many patients, but did change in only two patients. Patients who previously did not require premedication but whose cardiac status had changed such that premedication became necessary could have developed infective endocarditis if invasive dental procedures had been performed according to the original instructions. Dental professionals can only ascertain a young patient’s cardiac status by consulting their physician or obtaining information from their guardian. Although it was a rare case, dental practitioners should keep in mind that even low-risk patients may experience changes in their condition over time, and therefore make sure to ask about the cardiac status in regular dental check-ups and ideally write a letter of inquiry to the attending physician every few years.

Sublingual immunotherapy may require temporary interruption during surgical procedures, such as tooth extraction [19]. In 2023, the Japanese Society of Pediatric Dentistry issued a statement regarding pediatric dental treatment during sublingual immunotherapy, and recommended that consultation with the physician should be undertaken for dental procedures [20]. In the present study, there were only two letters of inquiry regarding patients undergoing sublingual immunotherapy. Interestingly, one response indicated conditional continuation of treatment, while the other response indicated discontinuation. It is unclear whether this difference was due to the patients’ conditions or the opinions of their physicians. Therefore, the primary physician should be consulted for patients undergoing sublingual immunotherapy to confirm the appropriate approach during dental procedures. The use of sublingual immunotherapy has been increasing in recent years, and dental professionals may encounter patients requiring sublingual immunotherapy more frequently [21]. Additional large-scale studies in this field are needed in the future.

To date, no adverse events have occurred among the participants in this study, who have undergone dental treatment in accordance with their physicians’ responses. Additional surveys are necessary to examine outcome by diseases in the future. It is also important for pediatric patients with systemic disease to focus on preventing oral diseases. Lile et al. performed an observational cross-sectional study of 202 schoolchildren and suggested that school-based prevention programs may improve the oral health status of populations [22]. In addition, our previous study showed continual dental support improved the periodontal condition and oral health habits of hemophilic patients [23]. These results also underscore the importance of preventive approaches in pediatric patients, where adequate oral care may help reduce the risk of complications and support overall health.

The present study has some limitations. First, this study is an exploratory investigation, and the sample size was small; therefore, methodological biases exist. Although we extracted referral information for a 5-year period, we found only a total of 48 letters of inquiry, and the classification of systemic conditions became broad. Increasing the sample size in future studies may enable comparisons focusing on specific disease entities. Further collaborative research with other medical institutions is essential. Second, the study was conducted at a university hospital, and the analysis included only pediatric patients who required an interdisciplinary consultation, excluding simpler cases or those managed without formal medical correspondence. Thus, the treated patients may have had more severe systemic conditions than those at a private dental clinic, and there may also have been selection bias. It is necessary to conduct similar surveys in private dental clinics in the future.

## 5. Conclusions

During dental treatment, attention must be paid to various systemic diseases, as the treatment types and approaches are constantly changing. Therefore, dental professionals must continually update their knowledge. Furthermore, since oral management of pediatric patients may require dental trauma treatment or primary tooth extraction, it is crucial to consult the patient’s physician before taking any action. Systemic conditions can change over time, potentially requiring different approaches. This study is of an exploratory and descriptive nature, and the limited generalizability of its findings. However, there were a few patients whose response from the patient’s physician changed at reconfirmation. Regular confirmation with the patient’s physician, rather than a single check, can help to establish a safer healthcare system for pediatric patients.

## Figures and Tables

**Table 1:** Changes in letters of inquiry over time.

Year	2021	2022	2023	2024	2025	Total
Male	2	6	4	6	3	21
Female	2	5	2	7	11	27
Total	4	11	6	13	14	48

Among the total 48 letters of inquiry, 5 letters involved the same patients (one male patient and four female patients).

**Table 2:** Ages and aims at the time of letter of inquiry creation.

Age (Years)		0–5	6–8	9–11	12–15	16–	Total
First confirmation		12	12	7	1	2	34
Aim	Oral management	5	2	1	0	1	9
	Caries treatment	5	3	2	1	1	12
	Surgical procedure	2	7	4	0	0	13
Reconfirmation		1	2	6	4	1	14
Aim	Oral management	1	2	3	0	0	6
	Caries treatment	0	0	0	1	0	1
	Surgical procedure	0	0	3	3	1	7
Total		13	14	13	5	3	48
Aim	Oral management	6	4	4	0	1	15
	Caries treatment	5	3	2	2	1	13
	Surgical procedure	2	7	7	3	1	20

Among the total 48 letters of inquiry, 5 letters involved the same patients (one male patient and four female patients).

**Table 3:** Physician responses to first confirmation letters.

Systemic Diseases			Physician Responses
Diseases of the circulatory system	24 (70.6%)	17 (70.8%)	Premedication with antibiotics for invasive dental procedures
		7 (29.2%)	No premedication required
Diseases of the nervous system	3 (8.8%)	1 (33.3%)	Caution regarding lower limb restraint
		2 (66.7%)	None
Diseases of the immune system	2 (5.9%)	1 (50.0%)	If sutures are placed during invasive dental procedures, discontinue sublingual immunotherapy until suture removal. If no sutures are placed, discontinue for 1 week postoperatively
		1 (50.0%)	Continue sublingual immunotherapy away from the surgical site during invasive dental procedures
Diseases of the ear or mastoid process	2 (5.9%)	1 (50.0%)	Contraindications for the use of monopolar type electrosurgical units
		1 (50.0%)	None
Others	3 (8.8%)	2 (66.7%)	None
		1 (33.3%)	When anxiety is severe, combine with visual aids
Total	34 (100.0%)		

**Table 4:** Physician responses to reconfirmation letters.

Systemic Diseases			Physician Responses
Diseases of the circulatory system	11 (78.6%)	7 (63.6%)	Premedication with antibiotics for invasive dental procedures
		1 (9.1%)	No premedication required → Premedication required
		1 (9.1%)	Premedication required → No premedication required
		2 (18.2%)	No premedication required
Others	3 (21.4%)	2 (66.7%)	Premedication with antibiotics for invasive dental procedures
		1 (33.3%)	Avoid pressure on the head
Total	14 (100.0%)		

An arrow indicates that the physician response has changed from the previous response. Two patients had two referral letters, resulting in duplication.

## Data Availability

The original contributions presented in the study are included in the article, further inquiries can be directed to the corresponding author.
